# Expression of the Fatty Acid Receptors GPR84 and GPR120 and Cytodifferentiation of Epithelial Cells in the Gastric Mucosa of Mouse Pups in the Course of Dietary Transition

**DOI:** 10.3389/fphys.2017.00601

**Published:** 2017-08-21

**Authors:** Patricia Widmayer, Soumya Kusumakshi, Franziska A. Hägele, Ulrich Boehm, Heinz Breer

**Affiliations:** ^1^Institute of Physiology, University of Hohenheim Stuttgart, Germany; ^2^Experimental Pharmacology, Center for Molecular Signaling (PZMS), Saarland University School of Medicine Homburg, Germany

**Keywords:** development, milk fat, stomach, fatty acid receptor, lipid sensing cells, ghrelin

## Abstract

During weaning, the ingested food of mouse pups changes from exclusively milk to solid food. In contrast to the protein- and carbohydrate-rich solid food, high fat milk is characterized primarily by fatty acids of medium chain length particularly important for the suckling pups. Therefore, it seems conceivable that the stomach mucosa may be specialized for detecting these important nutrients during the suckling phase. Here, we analyzed the expression of the G protein coupled receptors GPR84 and GPR120 (FFAR4), which are considered to be receptors for medium and long chain fatty acids (LCFAs), respectively. We found that the mRNA levels for GPR84 and GPR120 were high during the suckling period and progressively decreased in the course of weaning. Visualization of the receptor-expressing cells in 2-week-old mice revealed a high number of labeled cells, which reside in the apical as well as in the basal region of the gastric glands. At the base of the gastric glands, all GPR84-immunoreactive cells and some of the GPR120-positive cells also expressed chromogranin A (CgA), suggesting that they are enteroendocrine cells. We demonstrate that the majority of the CgA/GPR84 cells are X/A-like ghrelin cells. The high degree of overlap between ghrelin and GPR84 decreased post-weaning, whereas the overlap between ghrelin and GPR120 increased. At the apical region of the glands the fatty acid receptors were mainly expressed in unique cell types. These contain lipid-filled vacuole- and vesicle-like structures and may have absorptive functions. We detected decreased immunoreactivity for GPR84 and no lipid droplets in surface cells post-weaning. In conclusion, expression of GPR84 in ghrelin cells as well as in surface cells suggests an important role of medium chain fatty acids (MCFAs) in the developing gastric mucosa of suckling mice.

## Introduction

During the postnatal development of mice, the structure and function of epithelial cells lining the gastrointestinal tract undergo changes in accordance with the physiological requirements at the different developmental stages (Kataoka et al., 1984). For the stomach of newborn mice, it has been shown that the glandular buds are only rudimentarily developed with some enteroendocrine cells at their base and an unique epithelial cell type at the glandular surface (Keeley and Samuelson, 2010). The stomach mucosa remains immature during the entire suckling period and the small glands comprise of immature parietal and chief cells coinciding with low levels of acid and pepsin activity in the stomach (Keeley and Samuelson, 2010). During the late suckling period, at ~3 weeks of age, the structure of the stomach mucosa changes in preparation for weaning—the turning point in diet type—, i.e., when milk is successively replaced by solid food of lower digestibility (Chen and Fisher, 1983). During weaning, glandular morphogenesis continues to mature and the gastric glands reach a compartmentalized structure with active parietal cells and chief cells as well as an increasing number of enteroendocrine cells with distinct hormone profiles (Norlén et al., 2001; Karam et al., 2004).

Not only the digestibility but also the composition of the diet during the suckling phase (exclusively milk) and the post-weaning phase (solid food) is very different. In fact, during the suckling-weaning transition, the diet changes from high fat milk to protein- and carbohydrate-rich food. The energy contained in mouse milk is mainly due to the high fat content, which is characterized by fatty acids of different carbon chain lengths ranging from 4 to 18 (Hahn and Koldovský, 1966; Görs et al., 2009), but primarily of medium chain fatty acids (MCFAs) with carbon chain lengths from 6 to 12 and long chain fatty acids (LCFAs) with a chain length of more than 12. For suckling mice, the MCFAs seem to be particularly important since they are highly hydrolysable and represent a rapidly absorbable source of energy (St-Onge and Jones, 2002). Thus, during the first 3 postnatal weeks, the stomach mucosa has to deal with nutrients within the milk, most notably fatty acids. Since the gastric processing of the ingesta as well as the regulation of food intake is dependent on the nutrient composition of the ingested food, it is a prerequisite that the gastric mucosa is capable to sense nutrients in the luminal content (Psichas et al., 2015). Therefore, we hypothesized that nutrient sensing in the stomach during the suckling phase strongly relies on receptors which recognize fatty acids and that fatty acid receptors may be less relevant later on when the mice ingest protein- and carbohydrate-rich food. Candidate receptors for MCFAs and LCFAs are the G protein coupled receptors (GPCRs) GPR84 and GPR120 (FFAR4), which respond to fatty acids with a chain length of C9–C14 and C14–C22, respectively (Hirasawa et al., 2005; Wang et al., 2006; Tanaka et al., 2008). Hence, in this study we analyzed the expression levels for the receptor types GPR84 and GPR120 in the gastric mucosa during different postnatal stages and characterized the cell types which express fatty acid receptors during the suckling phase and post-weaning.

## Materials and methods

### Animals and nutritional experiments

Studies were performed with C57/BL6J and GPR120-IRES-Cre/eR26-τGFP (Wen et al., 2011; Kusumakshi, 2014) mouse pups. For qPCR experiments 7, 14, 18, 21, and 28 days old mice were analyzed and for immunohistochemical comparisons 2- and 4-week-old mice (*n* = 4, each age). Pups were maintained under controlled temperature (20–22°C) and light conditions (12-h light, 12-h dark cycle; lights on at 7 a.m. and off at 7 p.m.) with *ad libitum* access to their nursing mother, standard chow (3.06 kcal/g, 58% from carbohydrates and 33% from protein; V1534-300 R/M-H, ssniff Spezialitäten GmbH, Soest, Germany) and water until day 23. At 24 days of age, mice were separated from their mother and kept on standard chow and water. For immunohistochemical comparisons, groups of 8 littermates were used. At postnatal day 14, the first 4 littermates were taken out (suckling phase), and 2 weeks later the remaining 4 (post-weaning phase).

### Ethical statement

The institutional and national guidelines for the care and use of laboratory animals according to the Society of Laboratory Animals (GV-SOLAS) were followed and the institutional internal review committee approved the work (V318/14 Phy). All experiments comply with the Principles of Animal Care, publication no. 85-23, revised 1985, of the National Institutes of Health and with the current laws of Germany.

### Tissue preparation

After removal of the storage compartment fundus, the stomach was opened along the greater curvature and washed in 1 × phosphate-buffered saline (PBS: 0.85% NaCl, 1.4 mM KH_2_PO_4_, 8 mM Na_2_HPO_4_, pH 7.4). For RNA isolation, the dissected corpus was immediately transferred into a collection tube, frozen in liquid nitrogen, and stored at −70°C until use. For immunohistochemical analyses, the dissected stomach was immersion-fixed in 4% buffered paraformaldehyde in 150 mM phosphate buffer (pH 7.4) for 2 h at 4°C followed by cryoprotection in 25% sucrose at 4°C overnight, then embedded in Leica OCT cryocompound tissue freezing medium (Leica Microsystems, Bensheim, Germany), and quickly frozen on liquid nitrogen. Longitudinal sections (4–8 μm) were cut on a CM3000 cryostat (Leica Microsystems, Bensheim, Germany) and collected on Superfrost Plus microslides (Menzel Gläser, Braunschweig, Germany).

### RNA isolation, cDNA synthesis, and qPCR

Initially, total RNA of frozen samples was prepared with the NucleoSpin RNA kit followed by mRNA isolation with the NucleoTrap mRNA kit according to the manufacturer's protocol (Macherey-Nagel, Düren, Germany). Subsequently, 80–100 ng mRNA was reverse transcribed using oligo(dT) primers and SuperScript III Reverse Transcriptase (Invitrogen, Carlsbad, CA, USA). RNA integrity of each sample was confirmed by the amplification of the housekeeping gene encoding the ribosomal protein L8 with intron spanning primers to verify successful DNA removal.

Real-time PCR experiments were performed as previously described (Widmayer et al., 2012). In brief, quantitative changes in mRNA levels were detected using the Light Cycler (Roche Diagnostics, Mannheim, Germany). The qPCR reaction mixture (10 μl) consisted of 2 × KAPA SYBR Fast qPCR Master Mix (Peqlab Biotechnologie, Erlangen, Germany) and primer sets. Relative amounts of GPR84 and GPR120 transcripts were normalized to the expression of L8 which remained constant in all samples. Each assay included (in triplicate): for GPR84 6–7.5 ng of each tested cDNA, for GPR120 and for L8 a 1:10 cDNA dilution and a non-template control reaction. The following qPCR protocol was used: 95°C for 2 min, 95°C for 15 s, 60°C (for GPR120), or 65°C (for GPR84) for 15 s, 72°C for 8 s with 45 cycles. Then, a melting curve analysis was included to ensure that only a single, specific amplicon had been produced. Amplification of only one product was additionally confirmed by agarose electrophoresis. LightCycler Software 3.5 (Roche Diagnostics) results were exported as tab-delimited text files and imported into Microsoft Excel for calculation of the expression ratios using the mean crossing points of target and reference genes from controls and samples. For the amplification of GPR84, GPR120 and L8 the following intron spanning primers were used: GPR84 primers, (nt 50–223 from GenBank accession number NM_030720; the expected size of PCR products, 174 bp) 5′-GAC TGC CCC TCA AAA GAC CTG C-3′ and 5′-GCC ACG CCC CAG ATA ATT GC-3′, GPR120 primers, (nt 700-822 from NM_181748; 123 bp) 5′-GTG CCG GGA CTG GTC ATT GTG-3′ and 5′-TTG TTG GGA CAC TCG GAT CTG G-3′, and L8 primers, 5′-GTG CCT ACC ACA AGT ACA AGG C-3′ and 5′-CAG TTT TGG TTC CAC GCA GCC G-3′ (nt 548–771 from BC043017, 224 bp, genomic contamination: 375 bp). Data from 2 independent gene expression profiling experiments were collected and yielded similar results.

### Histological analyses

Cryosections were air-dried, rinsed in 1 × PBS for 10 min and incubated with blocking solution (1 × PBS with 10% normal donkey serum (NDS), 0.3% Triton X-100) for 30–60 min at room temperature. Then, the blocking solution was replaced by the primary antibody diluted in 1 × PBS containing 10% NDS and 0.3% Triton X-100 at 4°C overnight. Antibodies were used in the following dilutions: rabbit anti-GPR84 (sc-99106; Santa Cruz Biotechnology, Santa Cruz, CA, USA) 1:100, chicken anti-GFP (ab13970, Abcam, Cambridge, UK) 1:1500, goat anti-chromogranin A (CgA) antibody (sc-1488, Santa Cruz Biotechnology) 1:200, goat anti-ghrelin (sc-10368; Santa Cruz Biotechnology) 1:1,000, goat-anti somatostatin (sc-7819, Santa Cruz Biotechnology) 1:1,000, and rabbit anti-TRPM5 (courtesy of T. Gudermann and V. Chubanov) 1:800. Specificity and use of the antibodies were documented elsewhere GPR84: Abdel-Aziz et al. (2015); GFP: van der Heijden et al. (2016); CgA: Gerbe et al. (2011); ghrelin: Caminos et al. (2003); somatostatin: Haid et al. (2012); TRPM5: Kaske et al. (2007). In addition, control experiments on consecutive tissue sections were performed in which the respective primary antibody was omitted. After washing in 1 × PBS, bound primary antibodies were visualized using appropriate secondary antibodies conjugated to Alexa 488, Alexa 568 or Cy3 (Invitrogen, Karlsruhe, Germany; 1:500 in blocking solution) for 2 h at room temperature. After three rinses for 5 min in 1 × PBS, sections were counterstained with 4′, 6-diamidino-2-phenylindole (DAPI)-containing solution (1 μg/ml in 1 × PBS, Sigma Aldrich, Schnelldorf, Germany) for 3 min at room temperature to visualize nuclei, then rinsed in water and finally mounted in Mowiol (Roth, Karlsruhe, Germany). No immunoreactivity could be observed when the primary antibodies were omitted.

Nile red staining was performed using a 1:100 solution of Nile red in 1 × PBS (1 mg/ml in aceton, Invitrogen) for 5 min at room temperature to visualize lipid droplets, then rinsed in 1 × PBS, and mounted in Mowiol.

### Microscopy and imaging

Immunofluorescence was examined and documented with a Zeiss Axiophot microscope (Carl Zeiss MicroImaging, Jena, Germany). Images were captured using a SensiCam CCD camera (PCO Computer Optics, Kelheim, Germany), adjusted for contrast in AxioVision LE Rel. 4.3 (Carl Zeiss MicroImaging, Jena, Germany) and arranged in PowerPoint (Microsoft) or Adobe Photoshop (Adobe Systems, San Jose, CA, USA).

### Cell quantification

Cell counting was conducted as previously described (Widmayer et al., 2012). Briefly, random sampling fields of longitudinal sections through the proximal corpus were selected where the immunopositive cells for GPR84, GPR120-τGFP, CgA, ghrelin, and somatostatin resided. To determine the number of cells, microscopic digital images of four to five consecutive sections from one animal were acquired by defining sampling fields of 355 × 265 μm. Immunoreactive cells were only counted when the nuclei were clearly visible by DAPI staining. The average cell counts of immunopositive cells from three to four animals were determined and data expressed as number of positive cells per defined area.

### Statistical analysis

Relative expression was calculated according to Livak and Schmittgen (2001): Relative expression = 2^−(Ct target−Ct ref)^. For determination of cell numbers, values were given as mean ± *SD*. Significant differences between the groups were analyzed by the unpaired *t*-test with GraphPad Prism (Graphpad Software, www.graphpad.com). Statistical significance was set at *P* < 0.05.

## Results

### Changes in expression levels of fatty acid receptors during postnatal development

**Figure 1:**
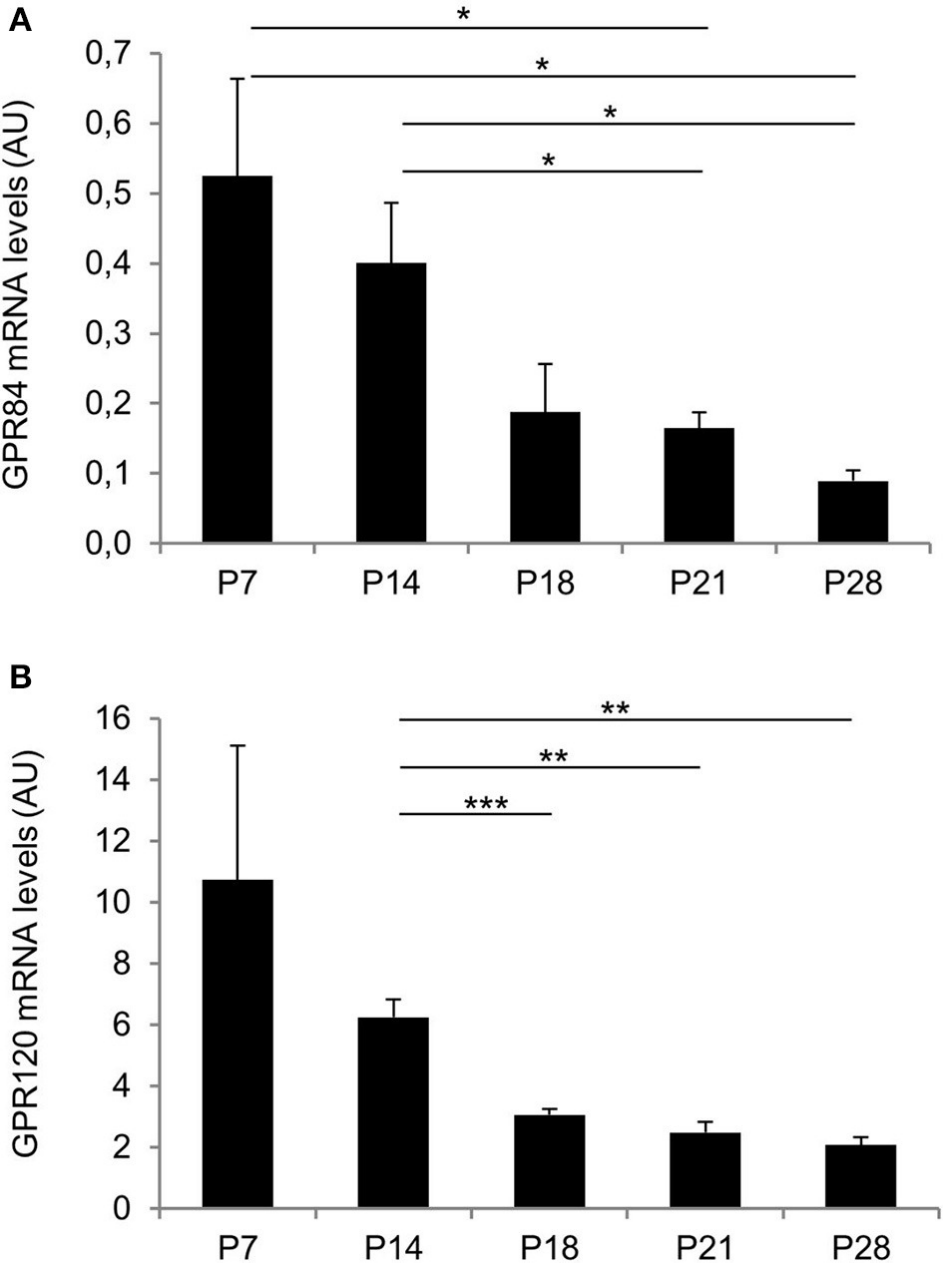
Changes in expression levels of GP84 and GPR120 in response to maternal and non-maternal diet. Relative expression levels for GPR84 and GPR120 were assayed by real time PCR from P7, P14, P18, P21, and P28. Each value corresponds to a pool of mRNA from 4 mice. Levels for both receptors were high at the suckling stages (P7, P14), declined with weaning (P18), and reached lower levels at P21 and P28 (post-weaning). Data were calculated using the comparative 2^−ΔΔCt^ method and expressed in arbitrary units (AU) as mean ± S.E.M. For normalization L8 was chosen that did not exhibit any significant change. Statistically significant results determined by the unpaired *t*-test are indicated by ^*^P < 0.05, ^**^P < 0.005, ^***^P < 0.0001.

Before weaning, mouse pups are exclusively nourished on milk with a high fat content exceeding 25% (Görs et al., 2009). Therefore, we hypothesized that receptors for fatty acids may be particularly important during the suckling period and thus be highly expressed in the gastric mucosa, whereas the expression levels may gradually decrease toward weaning. We first determined mRNA levels of the two fatty acid receptors—GPR84 and GPR120—by quantitative real time PCR analyses. The results depicted in Figure 1 indicate high levels of mRNA for both receptor types in 7 days old pups. Expression levels for both receptors remained high at 14 days of age, but then declined, as the pups began to be weaned and reached low levels at postnatal day 28 (for GPR84 AU: P7: 0.52 ± 0.14 and P14: 0.4 ± 0.09 vs. P21: 0.17 ± 0.02, *P* = 0.0294 and *P* = 0.0379, respectively, and vs. P28: 0.09 ± 0.02, *P* = 0.0139 and *P* = 0.0119, respectively; for GPR120 AU: P7: 10.72 ± 4.38 and P14: 6.25 ± 0.59 vs. P21: 2.48 ± 0.36, *P* = 0.0759 and *P* = 0.0015, respectively, and vs. P28: 2.09 ± 0.26, *P* = 0.0658 and *P* = 0.006, respectively; Figures 1A,B).

### Characterization of GPR84- and GPR120-expressing cells in the gastric mucosa at P14

**Figure 2:**
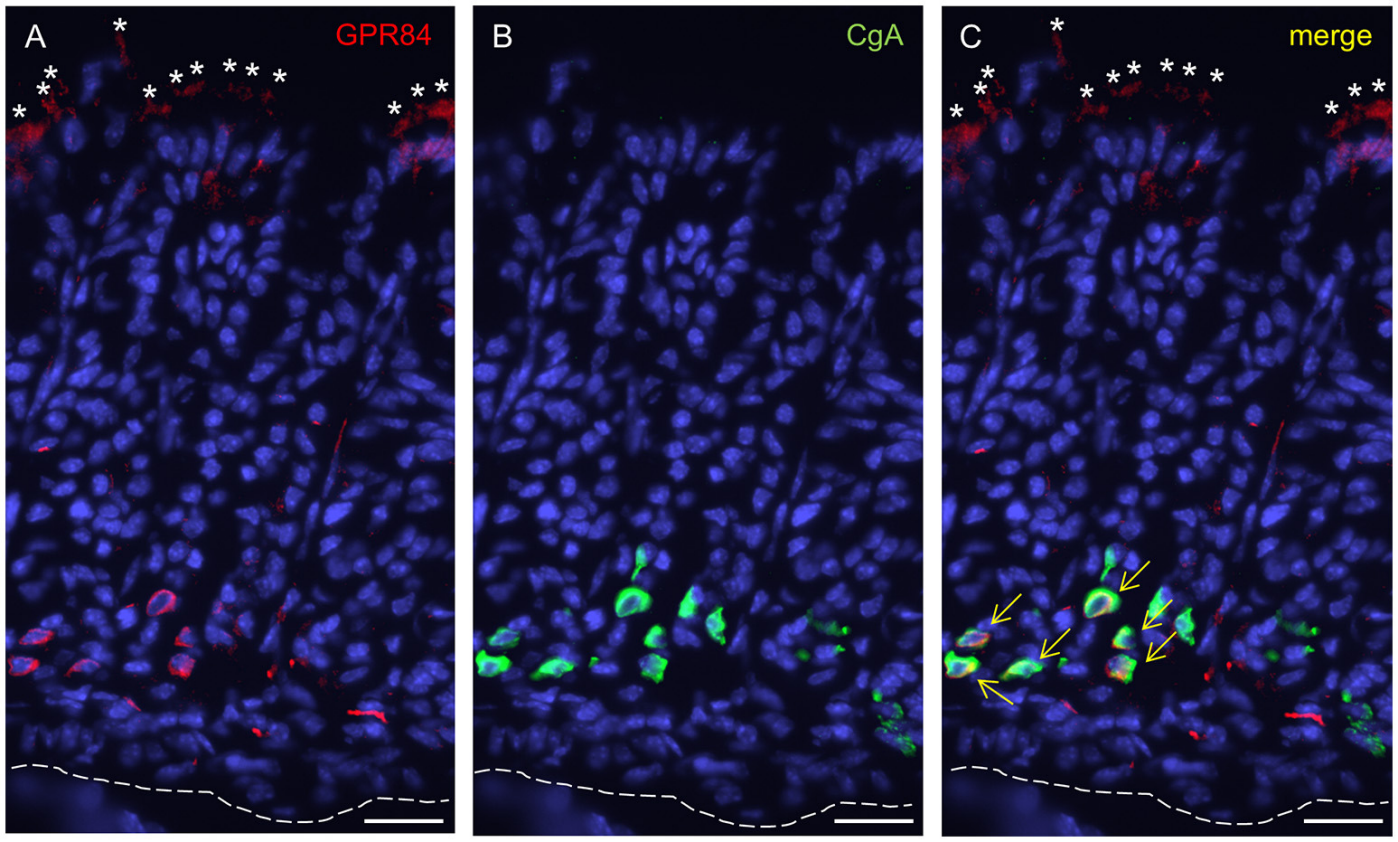
GPR84-expressing cells in the corpus mucosa of the mouse stomach at stage P14. **(A)** GPR84 immunoreactivity localizes to cells at upper and lower portions of gastric glands. (**B**) CgA-positive cells are located in deeper portions of the glandular invaginations. **(C)** Numerous CgA-positive endocrine cells were immunoreactive for GPR84 (yellow arrows). Asterisks point to apical poles of surface cells within the superficial folded epithelial lining, dashed line mark the transition between the mucosa and muscularis mucosae. Scale bar, 20 μm.

Based on the high expression levels for the two fatty acid receptors in the suckling period, we next determined the distribution and identity of cells which express GPR84 and GPR120 in the corpus mucosa of 2-week-old mice. For an immunohistochemical visualization of cells expressing GPR84, tissue sections were orientated perpendicular to the epithelial surface and probed with an antibody against GPR84. Immunoreactivity for GPR84 was visible in both the apical and the basal parts of the gastric glands of the developing gastric epithelium (Figure 2A). Labeled cells located at the glandular base morphologically resembled enteroendocrine cells, which are typically positioned in deeper regions of the glands (Figure 2B). These cells were also immunoreactive for chromogranin A (CgA), supporting the notion that they are endocrine cells (Figure 2C). At the apical region of the corpus glands, a rather diffuse staining for GPR84 was apparent (Figure 2A). The labeling occurred within large groups of tall slender and wide columnar- and goblet-shaped cells constituting the folded epithelial lining that covers the gastric glands (Figure 2A). More detailed analysis revealed that merely the apical poles of these cells were stained for GPR84.

**Figure 3:**
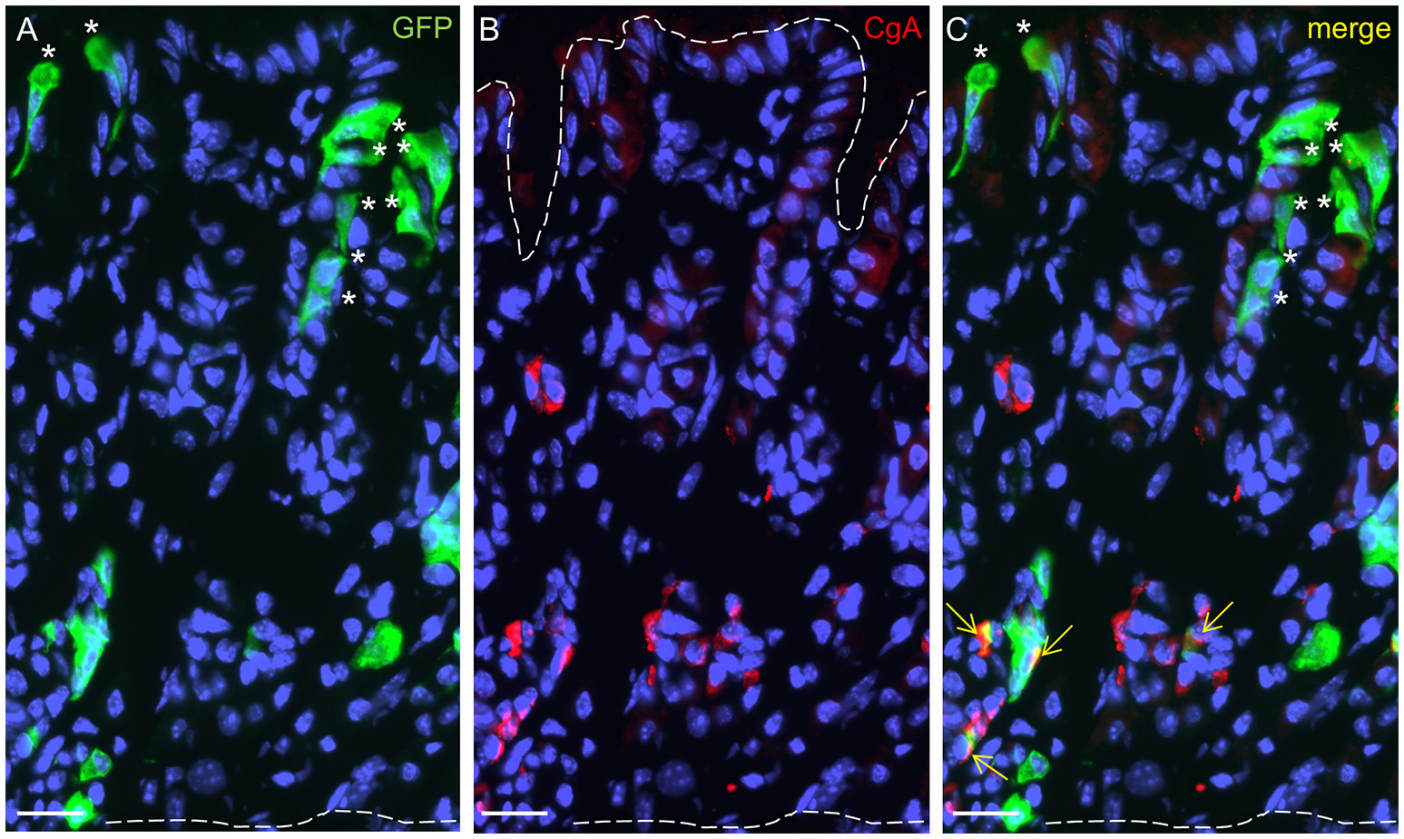
Localization and characterization of GPR120-τGFP-immunoreactive cells in the gastric glands at P14. **(A)** GPR120-τGFP cells are confined to basal and apical regions of glandular invaginations. **(B)** CgA positive cells reside in the lower half of the gastric mucosa. **(C)** Dual immunostaining demonstrates colocalization with CgA in a portion of endocrine cells (yellow arrows). Asterisks point to goblet-shaped epithelial cells at the surface and in the gastric pits, the upper dashed line marks the transition between mucosa and lumen, the lower one the transition between mucosa and muscularis mucosae. Scale bar, 20 μm.

In order to identify cells in the developing gastric epithelium that express the fatty acid receptor GPR120, a GPR120-IRES-Cre mouse strain was used that reports GPR120 expression by activating a τGFP transgene via Cre-mediated recombination (Kusumakshi, 2014). Immunolabeling for τGFP visualized cells that were particularly localized in the basal and the apical parts of the glandular invaginations (Figure 3A). At the glandular base, a subpopulation of GPR120-τGFP positive cells was also positive for CgA indicative for endocrine cells (Figures 3B,C). In addition, GPR120-τGFP immunoreactivity was further visible in numerous CgA-negative cells. These cells were more elongated and sometimes of triangular shape (Figure 3C). Besides invaginations with an apparent base and top distribution of GPR120-τGFP cells, some gastric glands displayed additional labeled cells which were scattered throughout the entire tissue (data not shown). These invaginations likely represent mucous glands reported to occur in distinct areas of the gastric mucosa (Lee et al., 1982). Within the surface epithelium, GPR120-τGFP immunoreactivity was confined to mostly small patches of goblet- and columnar-shaped cells. Single goblet-shaped GPR120-τGFP cells are visible in Figures 3A–C. In distinct areas, especially along the lesser and greater curvature, larger groups of surface cells were also labeled (data not shown).

**Figure 4:**
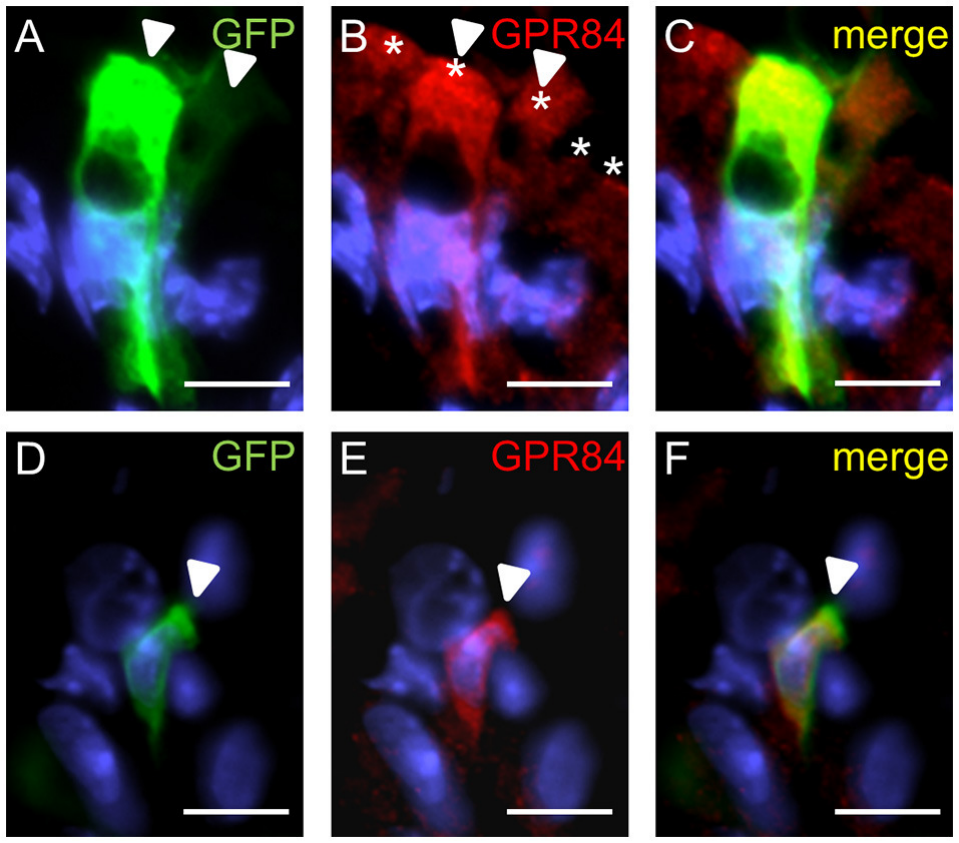
Visualization of distinct surface cell types expressing GPR120-τGFP and GPR84. **(A)** A goblet-shaped cell with a large oval apical vacuole-like structure and an adjacent slender columnar cell display different signal intensities for GPR120-τGFP (arrowheads). **(B)** A patch of GPR84-positive surface cells is marked by asterisks. **(C)** Merged image shows the localization of GPR120-τGFP cells belonging to a patch of GPR84-expressing surface cells. **(D–F)** A pear-shaped cell with an apical thickening (arrowhead) typical for brush cells is immunoreactive for GPR120-τGFP (**D**, green) and GPR84 (**E**, red). Scale bar, 10 μm.

**Figure 5:**
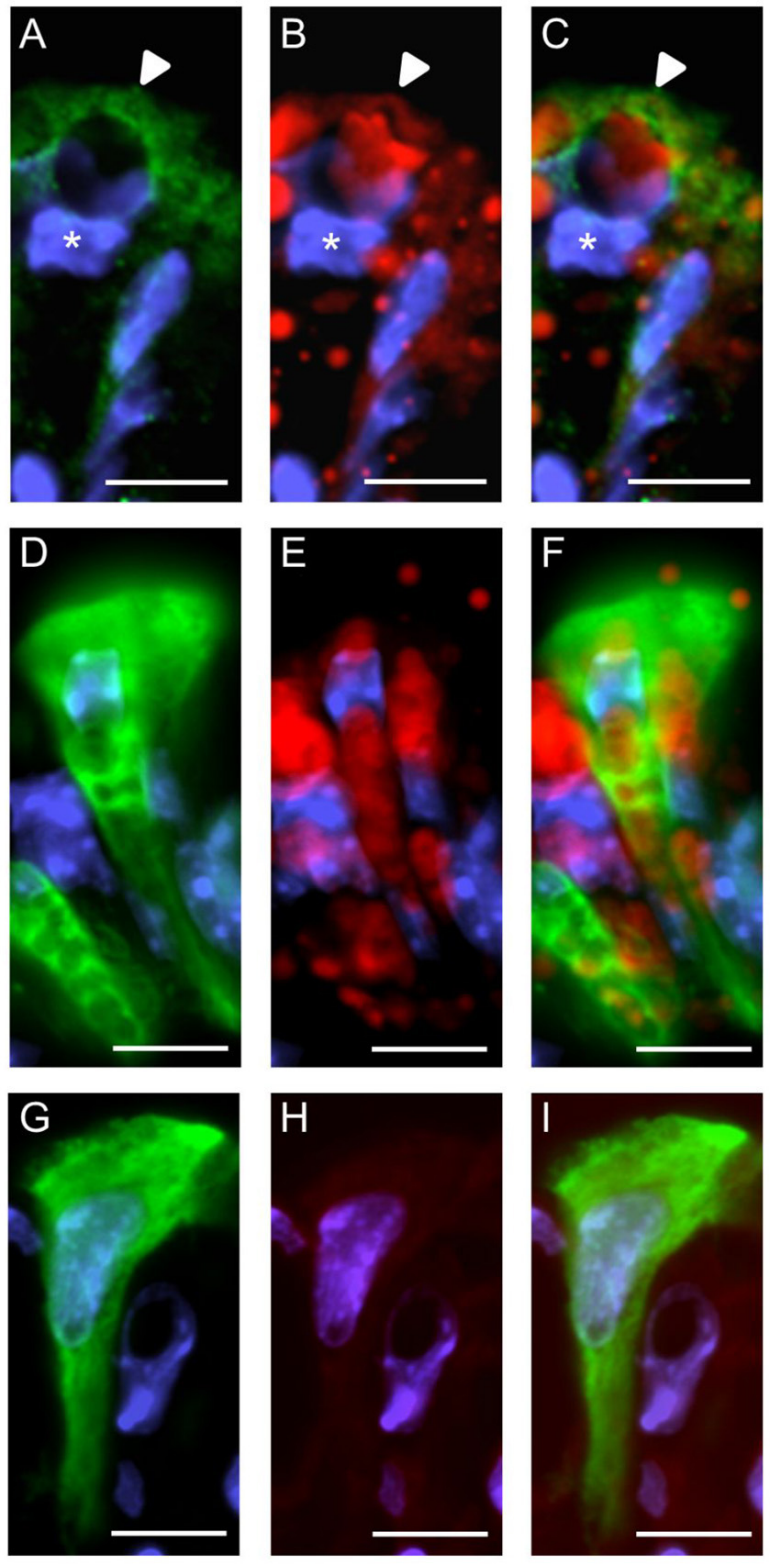
Characterization of GPR84 and GPR120-τGFP-expressing cells in the superficial epithelial lining at stage P14 and P28. **(A)** High magnification of a GPR84-positive cell revealing a surface cell type with large vacuole-like structures at P14. **(B)** Cytoplasmic content of surface cells is stained by Nile red. **(C)** Merged image shows that the vacuole is filled with lipid. Position of the nucleus is indicated by asterisks. **(D–F)** GPR120-τGFP positive surface cells with several vesicular- and small vacuole-like structures at P14. Labeling for GPR120-τGFP (**D**, green) and Nile red (**E**, red) shows staining of the cytoplasmic structures. **(G–I)** At P28, the superficial epithelial lining still comprises GPR120-τGFP positive surface cells (**G**, green), but is devoid of Nile red staining and shows a decreased immunoreactivity for GPR84 (**H**, red). Scale bar, 10 μm.

We then explored whether both GPCRs are coexpressed in the same surface epithelial cells. Double-labeling experiments for GPR120-τGFP and GPR84 revealed that some cells of the surface epithelium indeed expressed both fatty acid receptor types (Figures 4A–C). Analyses of the distribution patterns showed that GPR84 cells were mostly arranged in larger groups of surface cells. Some of these cells were also GPR120-τGFP positive indicating that a subpopulation expresses both receptor types (Figures 4A–C). While most of the double-labeled cells were columnar and goblet-shaped cells, very few cells were found that were small and pear-shaped instead (Figures 4D–F). The notion that these cells may represent brush cells was supported by colabeling with the brush cell marker TRPM5 (data not shown). One striking observation was an unique structural feature of the goblet- and columnar-shaped cells. They comprised small vesicle-like or large vacuole-like structures in their cytoplasm. The latter cell type seems to be only present in the superficial layer of the stomach of breastfed mice and to disappear with weaning and it has been speculated that these cells may be capable to absorb lipid from the luminal content (Helander and Olivecrona, 1970; Egelrud et al., 1971; Keeley and Samuelson, 2010). This view is based on the observation that the apical cytoplasmic content of these cells can be labeled by a lipid-soluble dye (Keeley and Samuelson, 2010). To analyze whether the vesicular structure in the cytoplasm of GPR84-positive surface cells might in fact be lipid droplets, sections were probed with GPR84 and Nile red. The results depicted in Figure 5A show, that a GPR84-immunoreactive cell comprises an oval cavity in its cytoplasm expanding above the nucleus. The content of this vacuole-like structure was stained by Nile red (Figures 5B,C). Among GPR120-τGFP cells only some contained large vacuole-like structures, while most of the cells contained smaller vesicular structures (Figure 5D). The content of these vesicles was also stained by Nile red suggesting that they are filled with lipid (Figures 5E,F). These results support the notion that goblet-shaped cells may also absorb dietary fats as previously proposed for surface cells with large vacuoles (Keeley and Samuelson, 2010). Consistent with a possible functional role of lipid-droplet containing surface cells in suckling stages, they disappeared with weaning as reported previously (Helander and Olivecrona, 1970; Egelrud et al., 1971; Keeley and Samuelson, 2010). Accordingly, no Nile red staining was visible in weaned mice and immunoreactivity for GPR84 was also decreased in the superficial layer (Figures 5G–I).

### Quantification of GPR84- and GPR120-τGFP-positive endocrine cells at P14 and P28

**Figure 6:**
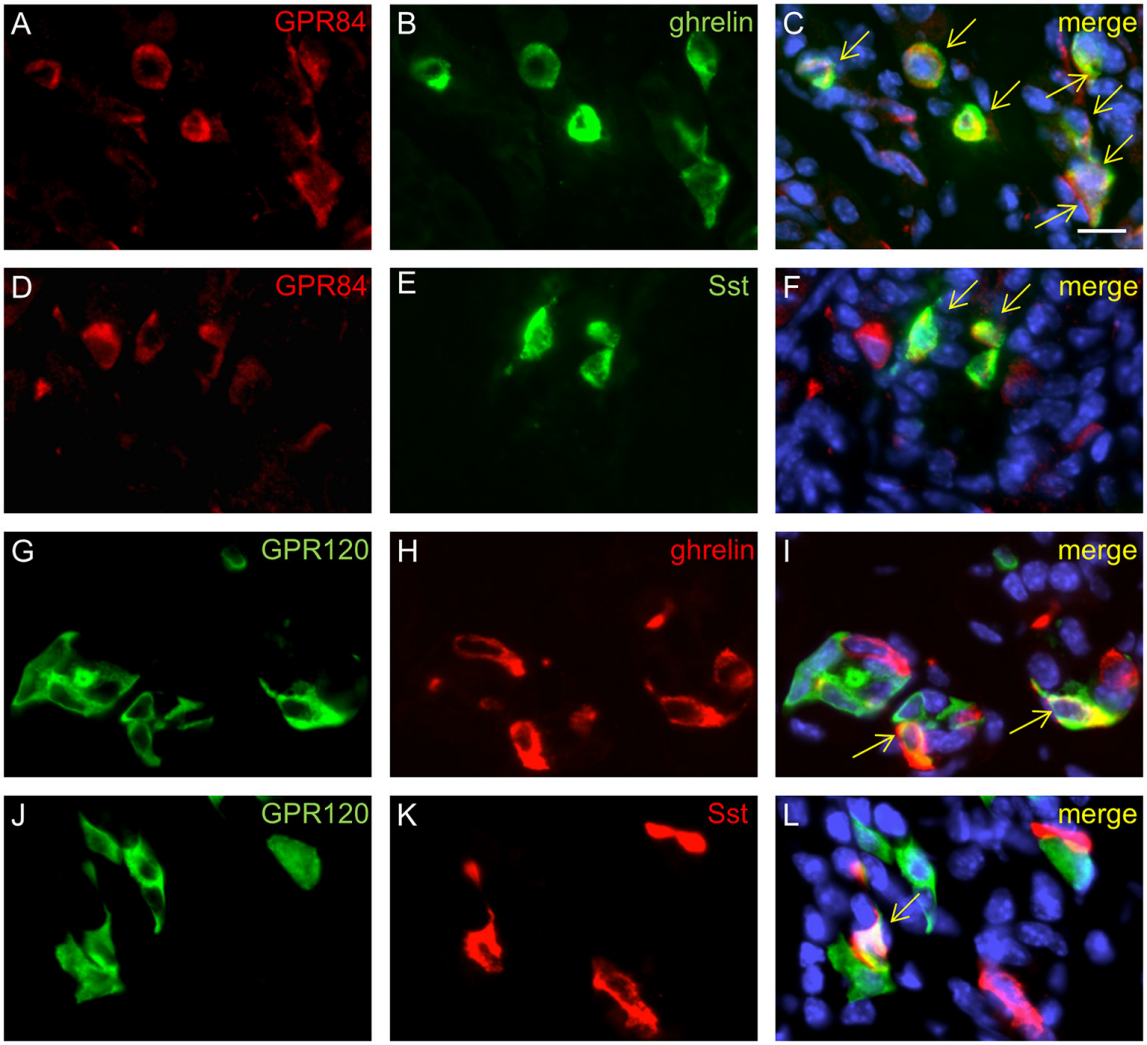
Characterization of GPR84 and GPR120-τGFP endocrine cells in suckling mice. Immunostaining for GPR84 (**A**, red) and ghrelin (**B**, green) demonstrates colocalization in all X/A-like cells (**C**, merge). Labeling for GPR84 (**D**, red) and somatostatin (**E**, green) shows coexpression in a subpopulation of GPR84 positive cells (**F**, merge). A subpopulation of GPR120-τGFP cells (**G**, green) displays immunoreactivity for ghrelin (**H**, green), as shown in the merged image (**I**). Single cells displayed immunoreactivity for GPR120-τGFP cells (**J**, green) and somatostatin (**L**, red), as shown in the merged image (**L**). Yellow arrows point to colabeled cells. Scale bar, 10 μm.

**Figure 7:**
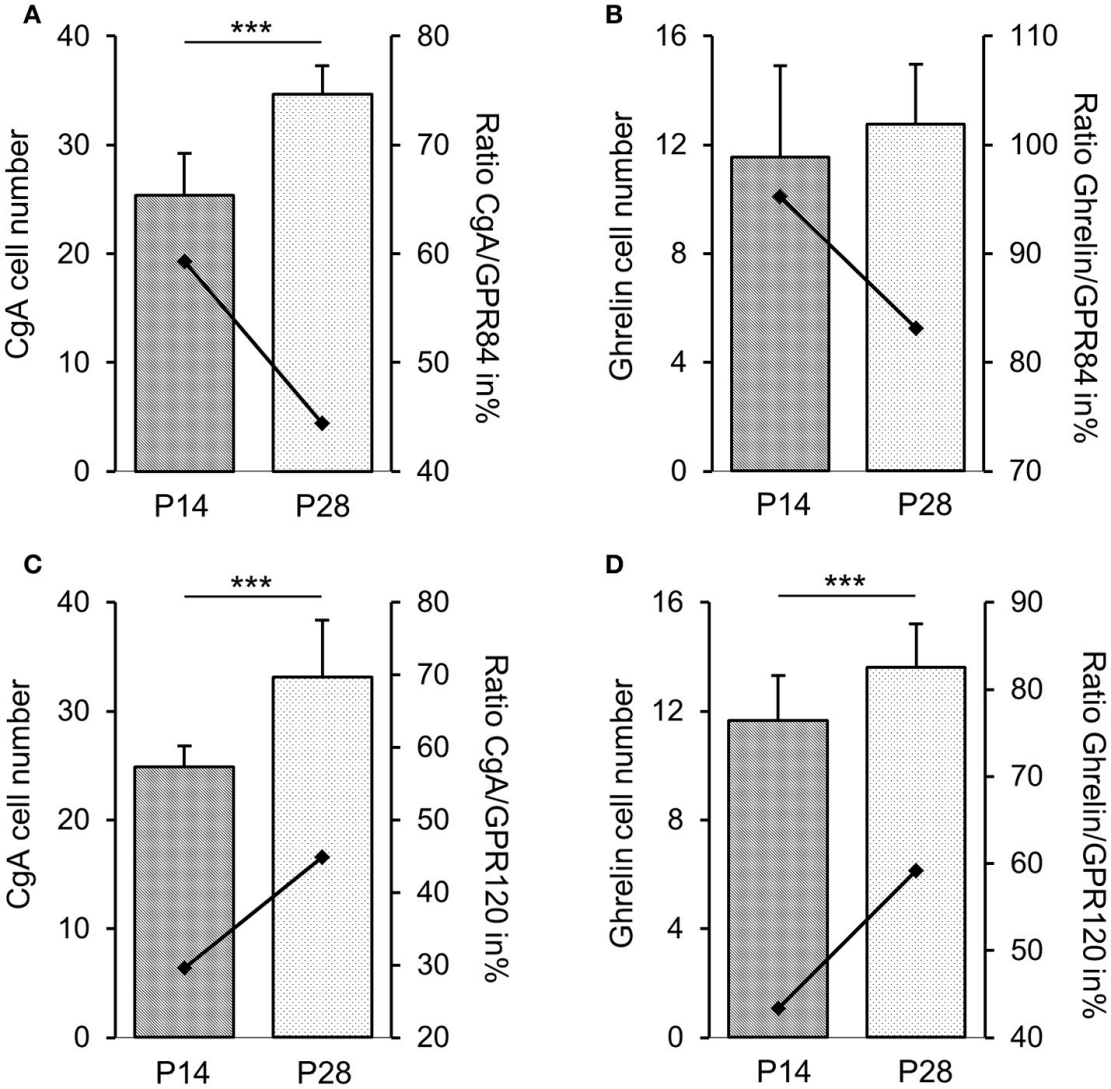
Changes in densities of endocrine cell types at stage P14 and P28. Frequencies of cells immunoreactive for CgA **(A,C)** and ghrelin **(B**,**D)** increased in the corpus mucosa. With maturation, the percentage of colocalization of GPR84 with CgA **(A)** and ghrelin **(B)** declined from 59 to 44% and from 95 to 83%, respectively. For GPR120, the percentage of overlap with CgA **(C)** and ghrelin **(D)** increased from 30 to 45% and from 43 to 59%, respectively. Cell densities are expressed as labeled cell numbers per unit area of 5 sections each animal (*n* = 3–4). Ratios are expressed in percentages (secondary axis). Mean numbers (±*SD*) of immunopositive cells at P14 (hatched) and P28 (dotted), ^***^P < 0.0001.

Previous transcriptome analyses indicated that GPR84 and GPR120 are expressed in X/A-like ghrelin-producing cells and somatostatin-secreting D cells (Engelstoft et al., 2013; Egerod et al., 2015). Thus, we finally determined whether fatty acid receptor-expressing cells colocalize with these endocrine cell types. In 2-week-old mice, immunostaining for ghrelin, accounting for almost half of the CgA-positive cells, revealed an almost complete overlap with GPR84 (Figures 6A–C), but less than one-half of the ghrelin cells colocalized with GPR120-τGFP (Figures 6G–I). Labeling for somatostatin revealed that some, but not all somatostatin cells showed immunoreactivity for GPR84 and GPR120-τGFP (Figures 6D–F,J–L). Due to the great overlap of GPR84 with CgA and ghrelin in suckling mice, we assessed the percentage of colocalization in the suckling stage and compared it to that of weaned mice. The analysis revealed robust changes in the frequencies of colabeling between the two developmental periods. Although the number of CgA and ghrelin cells increased during maturation, the percentage of colocalization of GPR84 with CgA and ghrelin declined (Figures 7A,B). In contrast, for GPR120 a marked increase in the percentage of overlap with CgA and ghrelin was observed during maturation (Figures 7C,D).

## Discussion

During the different stages of postnatal development of mice there is a fundamental change in the composition of ingesta. In contrast to the protein- and carbohydrate-rich solid food after weaning, the suckling mouse pups entirely depend on high fat milk. A high proportion of the triglycerides in murine milk contain MCFAs which seem to be particularly important for the pups since they represent a rapidly absorbable source for energy expenditure (St-Onge and Jones, 2002). The hypothesis that, due to the importance of the fatty acids during the suckling phase, the stomach mucosa of mouse pups may express receptor types for fatty acid sensing is supported by the results of this study. High levels of mRNA for the fatty acid receptors GPR84 and GPR120 were found in the suckling stages, which declined during weaning and reached low levels when weaning was completed. Although the functional role of fatty acid receptors in the stomach of suckling pups is elusive, it is conceivable that they may be involved in regulating fat-associated absorptive and/or secretory processes.

During the suckling phase staining for GPR84 appeared along the luminal surface of the stomach and was confined to numerous lipid droplet containing columnar- and goblet-shaped cells, GPR120 to some. This type of surface cell is only present during the suckling phase and supposedly directly absorbs the dietary lipids from the luminal content (Helander and Olivecrona, 1970; Keeley and Samuelson, 2010). Interestingly, it has been described that in suckling rats, especially the MCFAs are taken up by the stomach mucosa (Egelrud et al., 1971). Thus, the abundant expression of the receptor for MCFAs in surface cells coincides with the presumed absorptive properties of the stomach during the suckling phase. Moreover, surface cells with large lipid droplets also contain high concentrations of the fatty acid binding protein L-FABP during the suckling period and low levels in the weaning phase (Iseki et al., 1991). Although, it has to be clarified whether fatty acid receptors and binding proteins are in fact expressed in the same cell, one might speculate that an interplay of binding proteins and receptors might facilitate the uptake capacity for fatty acids by surface cells. The reduced level of fat in the ingesta after weaning implies a diminished absorption of lipid by surface cells in the stomach. This notion is in accordance with the observation that lipid droplets and labeling for the fatty acid receptor GPR84 in surface cells almost disappeared after weaning. These results are in line with the finding that surface cells with large lipid droplets are replaced by Muc5A producing cells during the weaning period (Keeley and Samuelson, 2010).

The results of our study indicate that in the suckling phase the ghrelin X/A-like cells account for nearly half of the enteroendocrine cells in the stomach and almost all of them express the receptor for medium chain fatty acids GPR84. This observation is in line with the notable role of MCFAs for the nutrition of mouse pups in the suckling phase, since they are highly hydrolysable and represent a major source for energy expenditure (St-Onge and Jones, 2002). It is therefore conceivable that the concentration of MCFAs in the gastric lumen may be an important parameter for controlling the secretion of the hormone ghrelin, which is an essential molecular signal for initiating food intake and inducing the release of growth hormone (Kojima et al., 1999; Nakazato et al., 2001; De Vriese and Delporte, 2007). Clearly, for such a scenario it is essential that X/A-like cells are capable to sense the concentration of MCFAs in the luminal content by having the appropriate receptor type. In this context, it is interesting to note that recent studies demonstrated that MCFAs can specifically affect X/A-like cells and lead to increased levels of acylated ghrelin in the stomach (Nishi et al., 2005; Janssen et al., 2012). The notion that the effects of MCFAs on ghrelin signaling are particularly important in the suckling phase is supported by the observation that in the post-weaning period the proportion of ghrelin cells, which express the receptor GPR84, is significantly declined, whereas the proportion of ghrelin cells, which express the receptor GPR120 for LCFAs, is increased. The change of overlap of receptor types in X/A-like cells during the weaning phase may foremost be an adaptive consequence of the greatly different composition of the solid food after weaning. In addition, the expression of GPR120 in X/A-like cells may have important functional implications, since LCFAs in the luminal content of the stomach cause a reduced level of ghrelin, an effect depending on the activation of GPR120 (Janssen et al., 2012; Lu et al., 2012; Gong et al., 2014). Thus, the effect of fatty acids on gastric ghrelin secretion might be dependent on the length of the hydrocarbon chain (Feltrin et al., 2006). Medium chain fatty acids enhance the secretion of ghrelin, while LCFAs diminish ghrelin release. The different choice of receptor types in X/A-like cells during the suckling phase (GPR84) and the post-weaning phase (GPR120) not only reflects changes in the composition of ingesta, but also points to some fundamental changes in the functional role of ghrelin signals in the course of the weaning process. In this context, it is worth mentioning that ghrelin has a dual action on both growth hormone secretion and food intake; it can therefore be considered as a bridge connecting somatic growth and body composition with energy metabolism (Camiña et al., 2003), aspects which are particularly relevant during the weaning process.

In conclusion, the transition from feeding exclusively on high fat milk to protein- and carbohydrate-rich solid food coincides with significant changes in the stomach mucosa including morphological modifications and variations in the repertoire of distinct cell types. At the molecular level—focusing in this study on receptors for fatty acids—there are distinct changes in the expression level but also in the receptor-expressing cell types; all the alterations seem to precisely match the physiological needs in the respective developmental stage.

## Author contributions

All authors listed have made a substantial, direct, and intellectual contribution to the work, and approved it for publication.

### Conflict of interest statement

The authors declare that the research was conducted in the absence of any commercial or financial relationships that could be construed as a potential conflict of interest.

## Abbreviations

CgA: Chromogranin A
DAPI: 4,6-diamidino-2-phenylindole
FFAR: Free fatty acid receptor
LCFA: Long chain fatty acid
MCFA: Medium chain fatty acid
qPCR: Quantitative polymerase chain reaction
TRPM5: Transient receptor potential cation channel, subfamily M, member 5.
